# When the Genome Plays Dice: Circumvention of the Spindle Assembly Checkpoint and Near-Random Chromosome Segregation in Multipolar Cancer Cell Mitoses

**DOI:** 10.1371/journal.pone.0001871

**Published:** 2008-04-02

**Authors:** David Gisselsson, Ulf Håkanson, Patrick Stoller, Dominik Marti, Yuesheng Jin, Anders H. Rosengren, Ylva Stewénius, Fredrik Kahl, Ioannis Panagopoulos

**Affiliations:** 1 Department of Clinical Genetics, Lund University Hospital, Lund, Sweden; 2 Department of Pathology, Lund University Hospital, Lund, Sweden; 3 The Nanometer Structure Consortium, Division of Solid State Physics, Lund University, Lund, Sweden; 4 Institute of Applied Physics, University of Bern, Bern, Switzerland; 5 Department of Clinical Sciences Malmö, Lund University, Lund, Sweden; 6 Department of Mathematics, Lund University, Lund, Sweden; Institute for Research in Biomedicine, Spain

## Abstract

**Background:**

Normal cell division is coordinated by a bipolar mitotic spindle, ensuring symmetrical segregation of chromosomes. Cancer cells, however, occasionally divide into three or more directions. Such multipolar mitoses have been proposed to generate genetic diversity and thereby contribute to clonal evolution. However, this notion has been little validated experimentally.

**Principal Findings:**

Chromosome segregation and DNA content in daughter cells from multipolar mitoses were assessed by multiphoton cross sectioning and fluorescence in situ hybridization in cancer cells and non-neoplastic transformed cells. The DNA distribution resulting from multipolar cell division was found to be highly variable, with frequent nullisomies in the daughter cells. Time-lapse imaging of H2B/GFP-labelled multipolar mitoses revealed that the time from the initiation of metaphase to the beginning of anaphase was prolonged and that the metaphase plates often switched polarity several times before metaphase-anaphase transition. The multipolar metaphase-anaphase transition was accompanied by a normal reduction of cellular cyclin B levels, but typically occurred before completion of the normal separase activity cycle. Centromeric AURKB and MAD2 foci were observed frequently to remain on the centromeres of multipolar ana-telophase chromosomes, indicating that multipolar mitoses were able to circumvent the spindle assembly checkpoint with some sister chromatids remaining unseparated after anaphase. Accordingly, scoring the distribution of individual chromosomes in multipolar daughter nuclei revealed a high frequency of nondisjunction events, resulting in a near-binomial allotment of sister chromatids to the daughter cells.

**Conclusion:**

The capability of multipolar mitoses to circumvent the spindle assembly checkpoint system typically results in a near-random distribution of chromosomes to daughter cells. Spindle multipolarity could thus be a highly efficient generator of genetically diverse minority clones in transformed cell populations.

## Introduction

In normal cells, mitotic cell division typically occurs in a bipolar fashion, resulting in two daughter cells with identical nuclear genomes. This restricted polarity is based on tight control of the centrosome cycle so that no more than two centrosomes are concurrently active during mitosis [1], [2]. However, in cancer cells, an excessive number of centrosomes can give rise to supernumerary spindle poles that may orchestrate a multipolar mitosis (MM), where the chromosome complement is pulled into three or more directions at anaphase [3], [4]. Since the first observations of MM in carcinomas by Hansemann in 1890 [5] multipolar spindles and centrosomal abnormalities have been reported in most common cancers [6]–[8]. Some studies have also indicated that MM may be a strong marker for adverse prognosis in tumor disease [9]–[11]. Furthermore, perturbations in centrosome number and structure have been linked to disturbed function of several cell cycle signaling pathways, such as inactivation of the TP53-, RB1- [12], [13], BRCA1- [14], [15], BRCA2- [16], and CDKN1A-proteins [17], as well as AURKA over-expression [18]. Through CCNE and PLK4, centrosomal disturbances have also been associated with exposure to viral carcinogens, most notably high-risk papilloma viruses [19], [20].

Considering the extensive information currently available on the molecular alterations causing spindle multipolarity in cancer cells, surprisingly little experimental data have been presented on the consequences of spindle multipolarity in transformed human cells. Previous studies have been confined to *in vitro* models of non-neoplastic cells from vole, mink, ox, and Rhesus monkey in which polyploidized cells progressed through multipolar cell division [21]–[24]. In these models, sister chromatids typically segregated through MM in haploid sets, resulting in euploid chromosome numbers in the daughter cells. This euploid segregation pattern has formed the basis for discussions on the role of MM in human tumours [25]. We now show that chromosome segregation in MM in human aneuploid transformed cells rarely, if ever, leads to segregation in the ratios expected from the principles of euploid segregation. Rather, a combination of an overall asymmetrical DNA distribution and circumvention of the spindle assembly checkpoint results in a near-random reshuffling of the chromosome complement.

## Results

### Experimental setup

The principles of the euploid segregation model implies that a mechanism exists which strictly regulates movement of a haploid set of chromosomes into daughter cells at mitosis [24]. Extrapolation of this theory to the typically aneuploid karyotypes observed in cancer cells means that each set of homologous chromosomes segregates as if the total chromosome number would be able to divide into distinct whole-number ratios. Thus, at least one copy of every chromosome will be present in each daughter cell, with exception of the sex chromosomes. In a cell division with two copies of a certain chromosome (disomic) dividing in three directions (tripolar), this would infer a segregation pattern through which two daughter chromosomes segregated to one of the daughter cells, while one daughter chromosome segregated to each of the other two daughter cells (*i.e.* a 2-1-1 segregation). The other possible disomic/tripolar segregation patterns (4-0-0, 3-1-0, and 2-2-0) would all result in at least one nullisomic daughter cell and would thus not be consistent with segregation in haploid sets.

**Figure 1:**
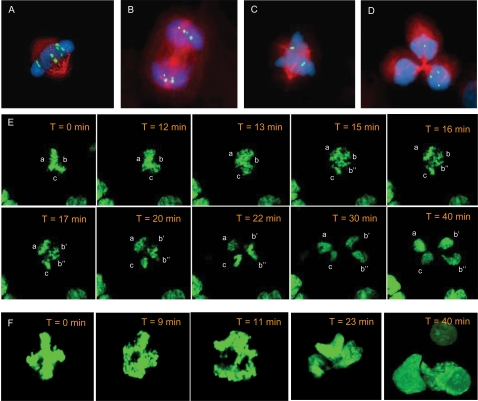
Chromosome dynamics. A chromosome 12 specific alpha-satellite probe (green) combined with immunofluorescence for beta tubulin (red) and DNA-counterstaining by DAPI (blue) shows tetrasomic/bipolar cell division at metaphase/early anaphase (A) and late anaphase (B) and a disomic/tripolar cell division at metaphase (C) and telophase (D); the segregation pattern is 4-4 in (B) and 3-1-0 in (D). Time-lapse microscopy of *H2B/GFP* transfected HEK293 cells shows succession of a tripolar metaphase configuration (E; poles denoted a-c) through two successive chromosome segregation events to four daughter nuclei and from a tetrapolar metaphase configuration (F) through a tripolar ana-telophase to three daughter nuclei shown by three-dimensional reconstruction of a confocal image stack at T = 40 min (T, time from first image).

To test whether segregation patterns of individual chromosomes at human multipolar cell divisions conformed to the principles of euploid segregation, we used two well studied cancer cell lines in which mitotic multipolarity has been associated with chromosomal instability (CIN), *i.e.* WiT49 from an anaplastic Wilms tumor with 7% MM [10], [26] and SW480 from a colorectal carcinoma with 4% MM [27], [28]. In order to compare cancer cell lines to non-neoplastic immortalized cells, we also included the adenovirus-transformed human embryonal cell line HEK293 with a complex karyotype and 1% MM [29]. In these cell lines, cross-labeling of DNA and spindle poles by beta-tubulin antibodies showed that the vast majority of multipolar cell divisions were either tripolar or tetrapolar, while <10% of MM had a higher polarity number (Figure 1A–D). In each cell line, the centromeres of two chromosomes which had showed little intercellular structural heterogeneity [10], [28] were labeled by fluorescence in situ hybridization (FISH): chromosomes 12 and 17 in WiT49, X and 18 in SW480, and 3 and 4 in HEK293, respectively. This selection was done to minimize confounding from anaphase bridging and other mitotic abnormalities primarily leading to abnormalities in chromosome structure. We then screened for the distribution of these chromosomes at anaphase in bipolar mitosis and MM.

### The euploid segregation model can be refuted

In total 15 490, 9 000, and 36 667 mitoses were screened in SW480, WiT49, and HEK293, respectively, and ana-telophase cells were selected for analysis of chromosome segregation (Table 1). To test the accuracy of the probe system we first screened morphologically normal bipolar ana-telophases, where 1∶1 segregation could be expected. All these cell divisions (2 323, 1 350 and 5 500 cells in WiT49, SW480 and HEK293, respectively) showed an equal number of chromosomes in the two daughter poles (Figure 1B). Sampling of multipolar anaphases was then performed, including disomic cells in SW480 and disomic as well as tetrasomic cells in WiT49 and HEK293. The reason for this selection was that in SW480 <10% of cells showed a copy number different than 2, but in WiT49 and HEK293 approximately 20% of cells were tetrasomic for the selected chromosomes. A total of 158, 54, and 49 analyzed multipolar anaphase cells in WiT49, SW480, and HEK293, respectively, were scored. With rare exceptions the multipolar cell divisions resulted in an unequal segregation of chromosomes among the daughter cells (Figure 1D). Summarizing the data for disomic/tripolar divisions, 2-1-1 segregation was most common (43%), followed by 3-1-0 (28%), 2-2-0 (23%), and 4-0-0 (6%) segregations. Among tetrasomic/tripolar cell divisions, the 3-3-2 segregation was the most frequent (22%), followed by 4-3-1 (20%), and 4-2-2 (18%), while the other configurations had frequencies <10%. Finally, for the disomic/tetrapolar configurations, the 2-1-1-0 configuration (42%) was the most common, while in the tetrasomic/tetrapolar cells the 4-2-2-0, 3-2-2-1, and 2-2-2-2 configurations (each at 19%) were the most common.

**Table 1:** Chromatid distribution in multipolar ana-telophase cells.

*Segregation patterns*	*Observed* 1	*Expected: obligate disjunction*	*Expected: obligate nondisjunction*	*Expected: random distribution*
**Disomy in tripolar mitosis**				
*HEK293 (chr 3, 4)*				
4-0-0	**4**	0	8	1
3-1-0	**9**	0	0	7
2-2-0	2	8	16	5
2-1-1	9	16	0	11
Sums	24	24	24	24
P^2^		<0.001	<0.001	ns
*WiT49 (chr 17)*				
4-0-0	**3**	0	22	2
3-1-0	**17**	0	0	19
2-2-0	18	22	43	14
2-1-1	27	43	0	29
Sums	65	65	65	65
P		<0.001	<0.001	ns
*SW480 (chr X ,18)*				
4-0-0	**1**	0	18	2
3-1-0	**14**	0	0	16
2-2-0	13	18	36	12
2-1-1	26	36	0	24
Sums	54	54	54	54
P		<0.001	<0.001	ns
**Tetrasomy in tripolar mitosis**				
*HEK293 (chr 3, 4)*				
8-0-0	0	0	1	0,01
7-1-0	0	0	0	0,2
6-2-0	**3**	0	7	0,6
6-1-1	0	0	0	0,6
5-3-0	**2**	0	0	1,3
4-4-0	1	1	6	0,8
4-2-2	4	6	11	4,8
4-3-1	4	7	0	6,4
5-2-1	**1**	0	0	3,8
3-3-2	10	11	0	6,4
Sum	25	25	25	25
P		Ns	ns	ns
*WiT49 (chr 12, 17)*				
8-0-0	**1**	0	2	0,03
7-1-0	**3**	0	0	0,4
6-2-0	**5**	0	18	1,5
6-1-1	**1**	0	0	1,5
5-3-0	**4**	0	0	3,1
4-4-0	7	2	13	1,9
4-2-2	11	13	27	11,5
4-3-1	13	18	0	15,4
5-2-1	**6**	0	0	9,2
3-3-2	9	27	0	15,4
Sum	60	60	60	60
P		<0.001	<0.001	ns
**Disomy in tetrapolar mitosis**				
*WiT49 (chr 12,17)*				
4-0-0-0	**1**	0	3	0,2
3-1-0-0	0	0	0	2,3
2-2-0-0	5	3	9	1,7
2-1-1-0	5	6	0	6,8
1-1-1-1	1	3	0	1,1
Sum	12	12	12	12
P		Ns	ns	ns
**Tetrasomy in tetrapolar mitosis**				
*WiT49 (chr 12,17)*				
8-0-0-0	0	0	0,3	0,001
7-1-0-0	0	0	0	0,03
6-2-0-0	**2**	0	3,9	0,1
6-1-1-0	0	0	0	0,2
5-3-0-0	**2**	0	0	0,2
5-2-1-0	0	0	0	1,3
5-1-1-1	0	0	0	0,4
4-4-0-0	2	0,3	3	0,1
4-3-1-0	2	2,6	0	2,2
4-2-2-0	4	2	11,8	1,6
4-2-1-1	**1**	0	0	3,2
3-3-2-0	0	0	0	2,2
3-3-1-1	0	5,3	0	2,2
3-2-2-1	4	7,9	0	6,5
2-2-2-2	4	3,0	2	0,8
Sums	21	21	21	21
P		Ns	ns	ns

1 Observations discordant with the obligate disjunction and obligate nondisjunction models are in bold and underlined type, respectively.

2 P values by the Chi-Square test.

Thus, in contrast to normal bipolar mitosis, MM typically resulted in an unequal copy-number of the studied chromosomes in daughter cells. Furthermore, most multipolar mitoses led to a significant proportion of cells with nullisomy in at least one of the daughter cells: 55% (78/143) of disomic/tripolar, 31% (26/85) of tetrasomic/tripolar, 92% (11/12) of disomic/tetrapolar, and 48% (10/21) of tetrasomic/tetrapolar mitoses, respectively. This refutes the hypothesis that multipolar cell divisions in transformed cell divisions can be modeled after the euploid chromosome segregation patterns found in non-transformed cells [25].

### DNA content in multipolar daughter cells is highly variable

**Figure 2:**
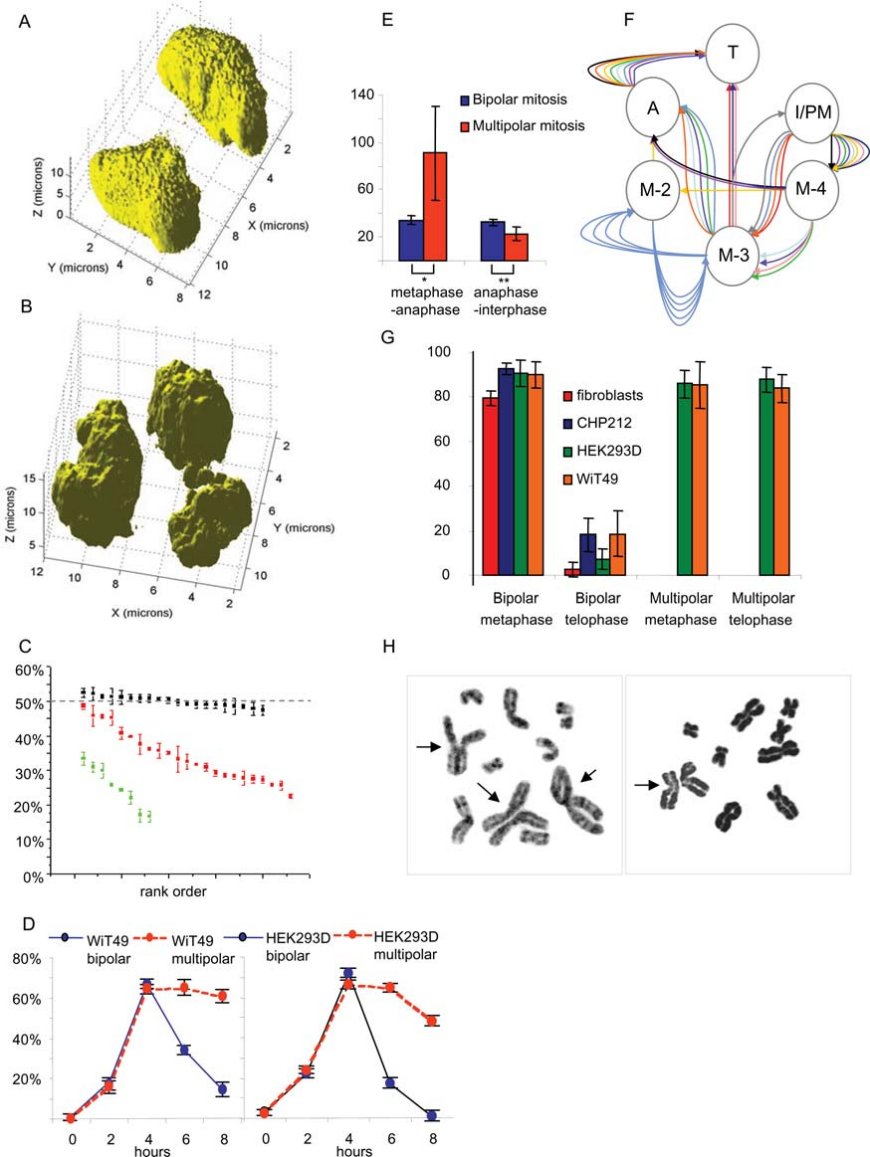
DNA distribution and timing. Reconstructed three-dimensional multiphoton cross sectioning images of a bipolar (A) and a multipolar (B) telophase cell in WiT49 show asymmetrical DNA-distribution in the latter configuration. Quantification of the relative amount of chromatin (C) in bipolar (black), tripolar (red) and tetrapolar (green) ana-telophase cells confirms a wide variation in the DNA content of multipolar daughter cells (X axis shows rank order according to DNA content). Measurement of the proportion of BrdU-positive bipolar (blue) and multipolar (red) mitotic cells at different time points after labelling (D) indicate delayed exit from mitosis for multipolar cell divisions in WiT49 and HEK293 (error bars indicate standard deviation). Time lapse imaging of H2B/GFP HEK293 cells show a prolonged metaphase-anaphase interval, and a reduced anaphase-interphase interval in multipolar compared to bipolar cell divisions (E); single and double asterisks indicate significance at the <0.05 and <0.01 levels respectively (Mann-Whitney U-test). One-per-minute time lapse imaging (F) of 12 multipolar mitoses, each corresponding to a flow of identically colored arrows, shows frequent polarity transformations (I = interphase, PM = prometaphase, M2 = bipolar metaphase, M3 = tripolar metaphase, M4 = tetrapolar metaphase, A = anaphase, T = telophase). Quantification of total intensity in arbitrary fluorescence units relative to centrosomal gamma tubulin fluorescence (G) shows a strong reduction of separase levels in telophase compared to metaphase in bipolar but not in multipolar cell divisions (error bars show standard deviation). Diplochromosomes (H, arrows) in G-banded partial metaphase spreads from WiT49.

To quantify the total distribution of DNA in multipolar ana-telophases, WiT49 was selected for further analysis because this cell line contained the highest frequency of MM. Previous studies of DNA-content in tumor cell mitoses have been performed by Feulgen staining in a two-dimensional setting [30]. However, MM often have a larger chromatin volume than normal mitoses with a diameter of up to 100 µm and a complex three-dimensional structure [31]. To be able to quantify the relative DNA-content of daughter ana-telophases under these conditions, we applied fluorescence quantification of DAPI-labeled DNA by multiphoton cross sectioning microscopy. This technique provides high spatial resolution imaging in a three dimensional setting due to the nonlinear signal generation at the laser focus [32]. The localized excitation greatly reduces out-of-focus fluorescence and photo-bleaching. To objectify the analysis further, a range of different thresholds for DAPI-intensity was chosen, extending from near the noise floor. For each threshold value, the ratio of the amount of fluorescence in each region to that in the first region was calculated. A mean relative DNA content for each ana-telophase pole was then calculated by averaging the results for all thresholds (Figure 2A–C).

The method was first tested on 20 methanol-fixed morphologically normal daughter ana-telophases from bipolar cell divisions. In these, the relative DNA content of individual poles compared to the total amount of chromosomal DNA in each cell division was approximately 50%, corresponding well to the expected 1∶1 segregation. We then evaluated daughter poles in tripolar and tetrapolar ana-telophases, respectively. Here the relative DNA content of daughter ana-telophases showed extensive variability, from 18–46% of the total cellular DNA content in tripolar divisions and from 16–34% in tetrapolar divisions. The fact that the DNA failed to segregate into stable, recurrent ratios further supported a non-euploid model of chromosome segregation and indicated a high degree of disorganization in these cell divisions.

### Multipolar metaphase is prolonged and undergoes polarity switches

The seemingly complex behavior of chromosomes in MM prompted us to investigate the time-course in multipolar compared to bipolar mitoses. To evaluate the total time spent in mitosis, we first labeled chromosomes in WiT49 and HEK293 cells with bromodeoxyuridine (BrdU). After cell cycle arrest by serum-starvation, BrdU was incorporated for 1 h in serum-rich medium after which cells were harvested every second hour. The mitotic cell pool that had been in S-phase during the labeling period was then detected by anti-BrdU-antibodies. Among the bipolar mitoses in both cell lines, the labeled cell pool made up the majority of dividing cells after 4 h, while very few labeled mitotic cells remained after 8 h (Figure 2D). Among MM, the labeled cell population also peaked after 4 h, but then remained to make up a large proportion of dividing cells even after 8 h. This indicated that MMs took substantially longer than bipolar cell divisions to exit mitosis.

To validate this finding we created a stable HEK293 transfectant expressing a histone-2B/green fluorescent fusion protein, allowing real-time monitoring of chromosomes during cell division (Movie S1) [33]. We then followed 10 bipolar and 12 multipolar cell divisions from prometaphase to the subsequent interphase and measured the intervals from the initiation of metaphase to metaphase-anaphase transition and from metaphase-anaphase transition to telophase-interphase transition. In bipolar divisions, the mean time from initiation of metaphase to the beginning of anaphase was 34 min (range 23–49), whereas in MM it was highly variable but overall considerably extended (P<0.05; Mann-Whitney U test) with a mean of 91 min (range 16–220 min; Figure 2E). In contrast, the time from the initiation of anaphase until interphase was slightly shorter in MM than in bipolar mitoses (mean 23 min compared to 32 min; P<0.01).

Of the 12 multipolar mitoses that were monitored, one did not segregate into daughter cells, but arrested in tripolar metaphase and then reverted to interphase (Figure 2F). Of the remaining 11 cells, four underwent either tripolar or tetrapolar mitosis without any shift in configuration. The other seven cells showed one or more polarity switches. One cell underwent chromosome segregation in two distinct steps, first going from tripolar metaphase to tripolar anaphase in which one of the three poles again divided, in total producing four daughter nuclei (Figure 1E; Movie S2). The other six cells shifted between various metaphase configurations before proceeding to anaphase (Figure 1F; Movies S3 and S4). Of the 11 cells that were followed from prometaphase through telophase, three did not show a clear separation of anaphase poles, seemingly passing from a complex metaphase configuration directly two telophase (Movie S5), with the time between these stages being 10 minutes or less. Because the time-lapse imaging was performed only in two dimensions, it cannot be excluded that at least some of the observed polarity shifts were due to rotations of the mitotic figures in Z-level, although no such rotations were evident. Nevertheless, our data showed that the dynamics of MM was distinctly different from bipolar mitosis, typically with longer metaphase duration in which polarity switches could occur.

### Sister-chromatid separation is unsynchronized in multipolar mitoses

**Figure 3:**
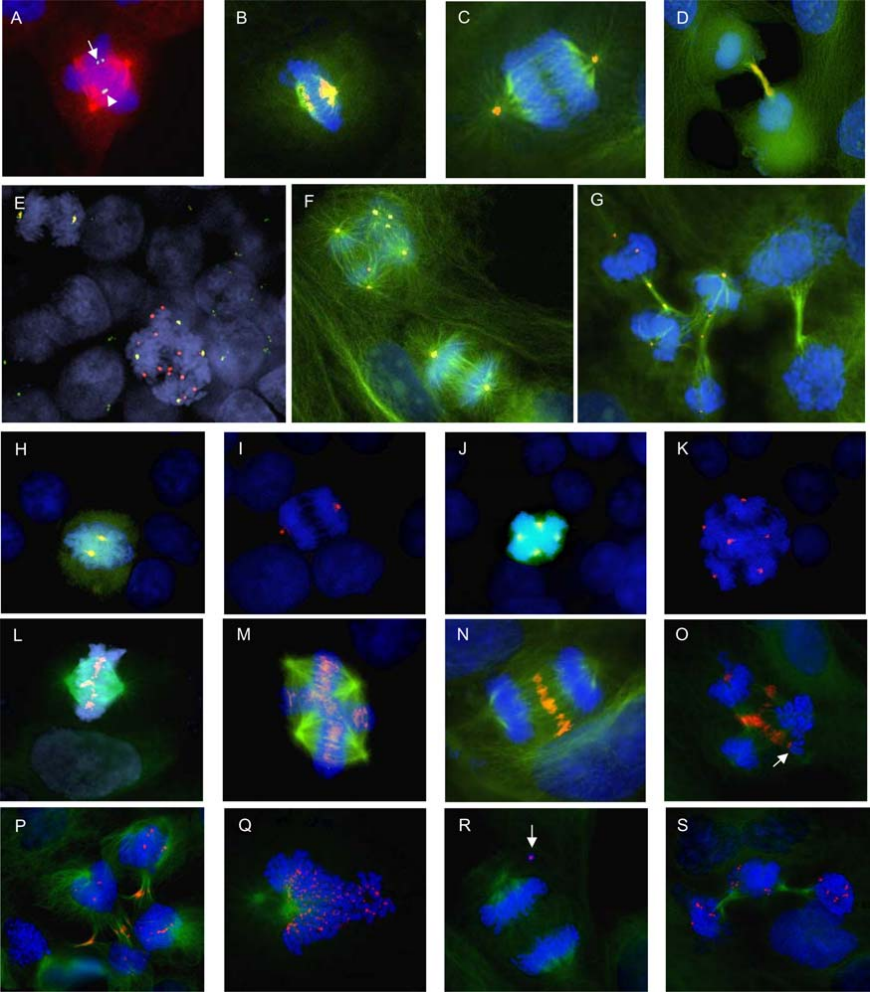
Sister chromatid separation and spindle assembly checkpoint proteins. FISH detection (A) of the chromosome 17 centromere (green) combined with immunofluorescence for beta tubulin (red) shows separation of the sister centromeres of one (arrow) but not of the other homologue (arrowhead). Immunofluorescence detection of separase (orange) and beta tubulin (green) shows localization of separase to spindle poles at metaphase (B) and early anaphase (C) and weak intensity staining in the midbody at telophase (D). Co-localisation of separase (red) and centrosomes (gamma tubulin in green) is observed at early bipolar anaphase (E, upper left) while several additional separase foci are present in an adjacent tetrapolar early anaphase cell (E, lower right). Co-labelling of separase (orange) and beta-tubulin (green) confirms this finding (F, tetrapolar at upper left and bipolar at lower right), and shows depletion of separase in a bipolar telophase cell (G, right) while several foci remain in a tetrapolar telophase cell (G, left). Immunofluorescence for CCNB1 (green) and AURKA (red) yields cytoplasmic staining in bipolar (H) and multipolar (J) metaphase cells while no staining is observed in bipolar (I) and multipolar (K) anaphase cells; AURKA stains the pericentrosomal regions at both metaphase and anaphase. Immunofluorescence for beta tubulin (green) and AURKB (red) shows localization of AURKB exclusively to centromeres at bipolar (L) and multipolar (M) metaphase, and exclusively to the spindle midzone at bipolar anaphase (N); in contrast, multipolar ana-telophase cells (O, P) exhibit AURKB in the midzone as well as on several chromosomes, indicating failure to separate the sister chromatids of some chromosomes (O, arrow). Immunofluorescence for beta tubulin (green) and MAD2L1 (red) shows MAD2L1 localization to centromeres at bipolar prometaphase (Q); at bipolar ana-telophase MAD2L1 foci is only present in chromosomes not incorporated in the mitotic process (R, arrow), whereas, at multipolar ana-telophase, multiple MAD2L1 foci are present on chromosomes (S).

The irregular and rapid transition from metaphase to telophase-interphase in MM, and the absence of clear anaphase configurations in some cells indicated that sister-chromatid separation might not occur in a regular fashion. To monitor sister chromatid separation in further detail in MM, we used the centromeric probe system described above and selected late metaphase/early anaphase cells for analysis in which at least one pair of sister centromeres in the metaphase/early anaphase plate had separated, as evidenced by split FISH-signals in a double-dot formation. In both WiT49 and HEK293, the vast majority (97% and 98%, respectively; Table 2) of the cells with a bipolar configuration in which one centromere had separated showed separation also of the other centromere(s), indicating a well coordinated metaphase-anaphase transition in these cells. In multipolar cells in WiT49 and HEK293, selected by the same criteria, less than half of the analyzed cell divisions were similarly coordinated. In fact, 59% and 55%, respectively, of these cells exhibited separation of only some of the probed sister centromeres while the others remained unseparated (Figure 3A). Sister chromatid in MM was thus often unsynchronized, compared to normal cell divisions.

**Table 2:** Unsynchronised centromere separation.

*WiT49*		
Unsynchronised	11	95
Synchronised	410	66
P^1^		<0.001
*HEK293*	Bipolar meta-anaphase	Multipolar meta-anaphase
Unsynchronised	5	28
Synchronised	280	23
P		<0.001

1 P values by the Chi-Square test.

### Multipolar metaphase-anaphase transition occurs by spindle assembly checkpoint slippage

To further investigate metaphase-anaphase transition in MM, we detected the intracellular localization of the ESPL1 (separase) protein during different stages of cell division. In mammalian cells, this protease is required for normal sister chromatid separation by cleavage of cohesin's kleisin subunit, but it is not necessary for other aspects of mitotic progression such as cytokinesis [34]. Human separase resides predominantly around the centrioles until late anaphase when it is degraded by an autocatalytic process triggered by its own activation of the anaphase-promoting complex [35]–[38]. Immunofluorescence (IF) detection with an antibody against the central part of separase (amino acids 1200–1300) in bipolar mitoses from WiT49, HEK293, and control cell lines without MM (fibroblasts and CHP212 neuroblastoma cells) accordingly resulted in fluorescence confined to the spindle poles at metaphase and early anaphase (Figure 3B and C). In bipolar metaphase, there were also occasional extra separase foci located to the mitotic spindle and overlapping with the location of chromosomes, similar to the distribution previously reported in yeast cells expressing a separase-GFP fusion protein [39]. In bipolar telophase cells, separase had disappeared from these locations and could only be observed in a minority (1–10%) of cells showing a faint signal at the midbody (Figure 3D). Further quantification of the total cellular separase fluorescence in 30 bipolar cell divisions from each cell line showed a substantial reduction at telophase, as expected from normal separase activation followed by autocatalytic degradation (P<3×10^−5^ in all cell lines, t-test; Figure 2G). Separase was then detected and quantified in multipolar metaphase and early anaphase cells from WiT49 and HEK293 (21 and 15 cells quantified, respectively). Similar to bipolar divisions, separase located to centrosomes in these divisions, but the extra-centrosomal fluorescence was more pronounced than in bipolar divisions (Figure 3E and F). In stark contrast to bipolar cells, multipolar telophase cells retained both centrosomal and spindle-located separase, and the total separase fluorescence was not reduced at this stage compared to metaphase (Figures 2G, and 3G; P = 0.83 and 0.37 in WiT49 and HEK293 respectively).

The incomplete degradation of separase in telophase cells indicated that multipolar cell divisions were able to bypass the spindle assembly checkpoint (SAC) that normally prevents metaphase-anaphase transition before all kinetochores are attached to opposite spindle poles. It is has been reported that the SAC in vertebrate cells does not arrest the mitotic process permanently. In fact, cells may eventually be driven through mitosis by a by a proteasome-dependent degradation of cyclin B (CCNB) [40]. To evaluate whether this phenomenon could explain why multipolar mitoses progressed through anaphase despite an incomplete separase cycle, CCNB1 and CCNB2 were detected by monoclonal antibodies in bipolar and multipolar mitoses in WiT49 and HEK293. Cells were cross-labeled with aurora kinase A (AURKA) antibodies in order to easily identify dividing cells. AURKA is localized predominantly in the pericentriolar region and expressed from the time of centrosome duplication at G2 until mitotic exit [41]. As expected, the vast majority of bipolar prometaphase and metaphase cells were strongly positive for CCNB1 and CCNB2, while bipolar anaphase cells were negative in both cell lines (Figure 2H and I; P<7×10^−15^ for pro/metaphase versus anaphase; Fisher's exact test; 69–197 cells scored in each cell line). Similarly, multipolar prometaphase and metaphase cells were positive, while all anaphase cells were negative for both CCNB1 and CCNB1 (Figure 3J and K; P<2×10^−4^; 20–55 cell scored).

To further test the hypothesis that MMs could pass from metaphase to anaphase by CCNB degradation despite a failure to completely satisfy the SAC, aurora kinase B (AURKB) and MAD2L1 were detected by immunofluorescence in WiT49 bipolar and multipolar mitoses. At normal metaphase, AURKB localizes to centromeres until bi-oriented attachment of kinetochores is obtained, after which it relocates to the spindle midzone in ana- and telophase [41]. It is not known precisely how this relocation is regulated, but it has been proposed that tension between bi-oriented sister chromatids sequesters AURKB in the inner centromere, thereby limiting the accessibility of this kinase to its substrates and relieving the spindle assembly checkpoint [42]. MAD2L1 locates to the kinetochores of chromosomes that have not yet bi-oriented, and delays anaphase onset by binding to and inhibiting CDC20 until all chromosomes are aligned on the metaphase plate; after normal metaphase-anaphase transition, MAD2L1 is no longer detectable at centromeres [43]. Accordingly, immunofluorescence for AURKB stained the centromeric regions of all the chromosomes in bipolar and multipolar WiT49 prometaphase and metaphase cells (Figure 3L and M) and the spindle midzone in the vast majority (97%) of bipolar anaphase cells (Figure 3N). In contrast, the majority (76%) of multipolar ana-telophase cells exhibited AURKB foci not only at the midzone but retained staining on some chromosomes (Figure 3O and P; P<1.1×10^−16^ for bipolar vs. multipolar; 189 ana-telophases scored). In some anaphase cells, the AURKB foci were clearly visible on chromosomes with unseparated sister chromatids (Figure 3O). As expected, MAD2L1 foci were detected on the centromeres in bipolar and multipolar prometaphase cells (Figure 3Q). In bipolar ana-telophase cells MAD2L1 foci were observed only in rare cell divisions with chromosome lagging (Figure 3R) and the majority (97%) of morphologically normal bipolar ana-telophases were MAD2L1-negative. In contrast, the majority (92%) of multipolar ana-telophases showed two or more MAD2L1 foci (Figure 3S; P<2×10^−17^ for bipolar vs. multipolar; 168 ana-telophases scored). Thus, MMs were able to degrade CCNB and exit mitosis without globally satisfying the SAC, as evidenced by retention of AURKB and MAD2L1 foci on some ana-telophase chromosomes.

### Capability of spindle assembly checkpoint slippage is not restricted to multipolar mitoses

To address whether the capability to circumvent the SAC was restricted to multipolar cell divisions, we exposed normal fibroblasts and WiT49 cells to the spindle-disrupting agent Colcemid for 18 h, after which the cells were stained with antibodies to AURKA and CCNB1. After Colcemid exposure of fibroblasts, the ratio of CCNB-positive metaphase cells to CCNB-negative ana-telophase cells (M/A) shifted dramatically from 1.8 (133/74) to 15.2 (214/14). In unexposed WiT49 the M/A ratio was 2.0 (528/258) for bipolar mitoses, while it was 5.9 (100/17) for MM (P<2×10^−5^ for bipolar vs. multipolar). The lower frequency of ana-telophase configurations among multipolar mitoses is consistent with metaphase being prolonged compared to anaphase in MM. After Colcemid exposure, the M/A ratio increased in both bipolar and multipolar WiT49 mitoses, to 50.3 (302/6) and 27.5 (110/4), respectively, with no significant difference between them (P = 0.47). Thus, a small fraction (2–6%) of mitoses in both fibroblasts and WiT49 cells were able to bypass the spindle assembly checkpoint irrespective of polarity, indicating that this checkpoint slippage mechanism is not unique for multipolar mitoses.

### Checkpoint slippage leads to frequent nondisjunction in multipolar mitosis

The finding that multipolar mitoses exited mitosis without complete separase activation and with retained chromosomal MAD2L1- and AURKB foci predicted that at least some chromosomes in the resulting daughter cells would consist of sister chromatids that remained attached at their centromeres. Such diplochromosomes have been described as a characteristic of separase-deleted cells and have been taken as evidence that separase is necessary for sister chromatid separation [34]. In metaphase-arrested chromosome spreads from WiT49, SW480 and HEK293, diplochromosomes were found in 5%, 5% and 2% of scored G-banded metaphase cells (65–100 in each cell line), respectively, whereas none were found in normal fibroblast cultures or in the neuroblastoma cell line CHP212 lacking multipolar cell divisions (>100 mitoses scored in each; Figure 2H). Taken together, these data indicated that chromatid separation did not occur in a regular fashion in MM and that this resulted in mitotic nondisjunction for at least some chromosomes.

**Figure 4:**
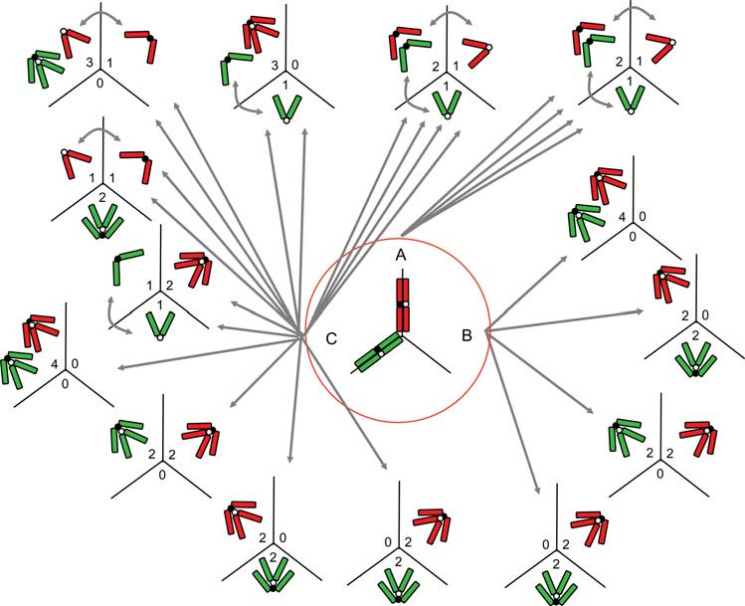
Models of sister chromatid separation. Segregation according to the total disjunction (A), total nondisjunction (B), and binomial random segregation (C) models exemplified for a disomic chromosome in a tripolar mitosis with the homologues positioned in different metaphase axes. Straight arrows indicate the number of possible segregation patterns according to each model, curved arrows indicate additional segregation patterns not drawn, red/green distinguishes the different homologues, and open/filled centromeric circles distinguish sister chromatids.

In order to investigate the frequency of nondisjunction in MM, we then compared the segregation patterns found in multipolar ana-telophase cells (Table 1; Figure 4) to three models of chromosomes segregation. The first of these postulated separation of all sister chromatids (complete disjunction), the second postulated no sister chromatid separation at all (complete nondisjunction), while the third model postulated that sister chromatid disjunction would be just as likely as nondisjunction (binomial chromatid segregation). Even though a total number of >60 000 cell divisions were scored in these experiments, only the disomic/tripolar category in all cell lines and the tetrasomic/tripolar category in WiT49 contained a sufficient number of counts to allow statistical testing. However, in all these cases the segregation patterns were significantly different from those expected from the first two segregation models, whereas the third model could not be refuted. Furthermore, in all categories of polarity, segregation patterns were observed that were incompatible with either one or both of the first two models (Table 1), *e.g.* 7-1-0 segregation in tetrasomic/tripolar mitosis, which can occur only if three of the four homologous chromosomes fail to undergo sister chromatid separation while the fourth undergoes separation. Taken together with the observations of a disrupted separase cycle, retained chromosomal AURKB and MAD2L1 foci at ana-telophase, unsynchronized centromere separation, and the finding of diplochromosomes, this strongly indicated that some sister chromatids separate regularly in MM whereas others do not, ultimately resulting in a segregation pattern which is not distinct from a random allotment of sister chromatids to the daughter cells.

## Discussion

The molecular mechanisms behind spindle multipolarity in human cells have been explored in detail during recent years [44]. A strong motive behind these studies is the presumed link between MM and the generation of somatic chromosome changes in tumor cells [3], [45], [46].. But for MM to contribute efficiently to aneuploidy their daughter cells must (1) contain a different number of chromosomes than the mother cell, going beyond the ploidy-level variation observed in normal cells, and (2) stand a good chance of proliferative survival *in vivo*. In the present study, the first of these two prerequisites was tested in three well-known *in vitro* systems. Our data unequivocally show that MM in aneuploid transformed cells does not lead to an organized chromatid separation in distinct sets, as was suggested by previous studies of untransformed animal cells [21]–[24]. In all of the three investigated cell lines, MM gave rise to a high proportion of cells completely lacking a copy of the probed centromere. This does not preclude the presence of sequences from the corresponding chromosome translocated to other chromosomes, but strongly speaks against any organization of centromeres into near-haploid sets at the metaphase-anaphase transition [21]–[24]. Our high-resolution measurements of the relative DNA content in multipolar daughter cells did not show recurrent segregation into whole number ratios. Furthermore, scoring the segregation patterns of individual chromosomes in a high number of ana-telophases showed that their segregation was significantly different from a situation in which all sister chromatids underwent separation, which is a basic prerequisite for segregation into haploid sets. In fact, we found that the segregation pattern in MM was best described by a model in which some sister chromatids separated while others did not.

In normal bipolar mitosis, the activation of the anaphase promoting complex after complete metaphase alignment leads to synchronized activation of separase by degradation of securin, followed by proteolytic cleavage of cohesin, which triggers concerted sister chromosome separation at the metaphase-anaphase transition. Segregation of unaligned chromosomes is prevented by the SAC. Through this checkpoint, degradation of securin and CCNB by the anaphase-promoting complex is inhibited by MAD2L1 at unattached kinetochores, in its turn leading to maintenance of high separase levels as this protein is prevented from activation and autocatalalytic cleavage. Our finding of a retention of chromosomal MAD2L1 foci and incomplete separase degradation at ana-telophase and the repeated attempts of MMs to form metaphase plates before finally undergoing metaphase-anaphase transition indicated prolonged activation of the SAC in these cell divisions. However, the vast majority of multipolar ana-telophases showed a reduction of CCNB levels, similar to bipolar ana-telophases, indicating that multipolar cells were able to eventually bypass SAC and exit mitosis. Such irregular metaphase-anaphase transition, before bi-orientation of all chromosomes, would readily explain our findings of unsynchronized sister-chromatid separation, a high frequency of non-disjunction, and diplochromosomes in metaphase spreads. The fact that similar SAC slippage was found also for bipolar cells in WiT49 and normal fibroblasts after prolonged Colcemid exposure is consistent with a previous study, showing that SAC-arrested mammalian cells may be driven through mitosis by a proteasome-dependent degradation of CCNB [40]. The finding that most multipolar metaphase cells ultimately exited mitoses and formed daughter cells therefore cannot be taken as evidence that the SAC is inherently defective in cells undergoing MM. Even though only a minority of bipolar mitoses were observed to bypass the SAC after Colcemid exposure, it is possible that the mechanism for SAC circumvention after prolonged activation by unaligned chromosomes is similar in bipolar mitoses and in MMs. The failure of multipolar cell divisions to achieve bi-orientation of all chromosomes, despite repeated attempts of metaphase plate formation, may thus be sufficient *per se* to drive these cell divisions out of mitosis by circumvention of the SAC.

Our observations corroborates that notion that spindle multipolarity may be efficient generators of aneuploidy in cell populations [3], [45], [46]. However, even if multipolar cell divisions may be allowed to progress through telophase, the potential of MMs to generate aneuploidy and clonal evolution in neoplastic tissue depends on the proliferative survival of multipolar daughter cells. This issue largely remains to be explored. One recent study on colorectal carcinoma cell lines indicated that cells having undergone MM rarely survive to form clones *in vitro* and, therefore, contribute less to clonal evolution than chromosome lagging and chromosomal breakage-fusion-bridge cycles [28]. Indeed, the finding of diplochromosomes in only 2–5% of metaphase spreads in the present study, indicates that non-disjunction in MM contribute to chromosomal aberrations only in a small minority of cells. However, under favorable micro-environmental conditions, even such a small fraction of surviving cells could expand to larger clones. The constant generation of novel numerical chromosome aberrations by MM could therefore have an important role in tumor development by facilitating a tumor's adaptation to micro-environmental cues or by promoting the development of resistance to chemotherapeutic drugs. This is consistent with several recent studies showing a strong correlation between centrosomal disturbances and/or spindle multipolarity, on the one hand, and poor response to treatment, on the other hand, for several tumor types, including urothelial cancer [9], [11], ovarian cancer [47], head- and neck-cancer [48], breast cancer [49], Wilms tumor [10], and multiple myeloma [50]. By defining the basic rules for chromosome segregation in multipolar cell division in transformed cells, the present investigation opens up for further exploration of the biological role of centrosomal defects in carcinogenesis.

## Materials and Methods

### Cell lines and culture

SW480 was obtained from the American Type Culture Collection and WiT49 was kindly donated by Dr.Yeger at the Laboratory of Medicine and Pathobiology, University of Toronto, Canada. The human embryonal kidney cell line HEK293 was obtained from The Banca Cellule e Colture in GMP, Genova, Italy. Cells were cultured in DMEM/F-12 medium (Gibco Co. Grand Island,. N.Y., USA), supplemented with fetal bovine serum (10%), 2 mM L-glutamine, 100 IU/ml penicillin, and 100 µg/ml streptomycin. Cell culture, harvest and chromosome preparation for banding and FISH were performed according to standard methods [51]. MM were scored as defined as by Jin et al. [9]. For BrdU labeling, cells were first cultured in RPMI1640 only (Gibco) for 48 h, after which serum and 10 µg/ml BrdU were added for 1h before cell cultures were harvested every second hour for 8 h, fixed in methanol, and subjected to labeling with murine anti-BrdU followed by anti-mouse-Cy3 antibodies (Amersham, Amersham Place, Little Chalfont, UK). Prolonged metaphase arrest was achieved by growth in Colcemid for 18 h at a final concentration 1 µg/ml as described [52].

### IF and FISH

Chromosome-specific centromeric probes were from Vysis Inc. (Downers Grove, IL). Beta-tubulin was detected by the murine monoclonal antibody TUB2.1 O95K4841 and gamma tubulin by the murine monoclonal antibody GTU-88 O26K4810 (Sigma-Aldrich, St. Louis, MO). The rabbit ab3762 antibody, directed against a synthetic peptide derived from residues 1200–1300 of human separase (Abcam, Cambridge, UK) was indirectly labelled by Cy3-conjugated anti-rabbit (Sigma). Cyclins B1 and B2 were labeled with the mouse monoclonal antibodies V152 and X29.2 (Abcam, Cambridge, UK), Aurora kinase A was detected by the polyclonal rabbit antibody A300-071A (Bethyl Laboratories Inc., Montgomery, TX), Aurora kinase B by A300-431A (Bethyl Laboratories Inc), and MAD2L1 by ab24588 (Abcam). Combined IF and FISH was performed as described with concurrent probe and target DNA denaturation at 95 °C for 10 min [53]. Sister centromeres were defined as separated if they displayed a double-dot configuration with at least one signal-width between them. Separase and gamma-tubulin fluorescence intensities were quantified in raw images by the Telometer software (http://bui2.win.ad.jhu.edu/telometer/).

### H2B-GFP transfection system

A genomic DNA fragment which encodes the full length H2B was amplified using the forward primer CGGGTACCGCCACC ATG CCA GAG CCA GCG AAG TCT G and the reverse CGG TGG ATC CCG CTT AGC GCT GGT GTA CTT GGT GAC. PCR amplification was performed in a 50 µL reaction volume containing 1x AccuPrime *Pfx* reaction mix, 1 unit AccuPrime *Pfx* DNA polymerase (Invitrogen, Carlsbad, CA), 0.3 µM of each of the forward and reverse primers and 300 ng genomic DNA. The PCR was run on a PCT-200 DNA Engine (MJ Research, Waltham, MA). The cycling included an initial denaturation at 95°C for 2 min, followed by 30 cycles of 15 s at 95°C, 30 s at 58°C, and 1 min at 68°C, and a final extension for 5 min at 72°C. The amplified fragment was double digested with *Kpn*I and *Bam*HI restriction endonucleases and cloned between the corresponding sites of a pEGFP-N1 vector (Clontech, Mountain View, CA), with the 5′end in frame with the cDNA coding for enhanced green fluorescence protein. The sequence was verified using the ABI Prism BigDye terminator v1.1 cycle sequencing kit (Applied Biosystems, Foster City, CA) and theApplied Biosystems Model 3100-Avant DNA sequencing system. As a transfection system, we used the Lipofect-AMINE 2000 reagent (Invitrogen Life Technologies, Carlsbad, CA) according to instructions provided by the manufacturer for HEK293 cells. After five days of growth in medium containing 1 µg/ml Geneticin, >75% of nuclei were GFP-positive.

### Multiphoton microscopy

The experimental setup consisted of a Coherent Mira Ti:sapphire ultra-short pulse laser (Santa Clara, CA, USA) and a custom-modified Zeiss Axiovert 100 TV (Carl Zeiss AG, Jena, Germany) inverted microscope. The output beam from the Mira (800 nm wavelength, 135 fs full-width at half-maximum pulses, 76 MHz repetition rate, and 10 nJ maximum pulse energy) was expanded and spatially filtered to ensure optimal focusing. The beam was then incident on a dichroic beamsplitter (Omega Filters XF2033, Brattleboro, VT, USA) designed to reflect light at the laser frequency and transmit shorter wavelengths. The reflected beam was raster scanned using a two-axis beam-scanning unit (GSI Lumonics, Billerica, MA, USA) and focused onto the sample by a Nikon 60X 1.2 NA water-immersion objective (mounted above the sample using custom-built hardware). The sample was placed on a piezoelectric translation stage (P562.3CL, Physik Instrumente, Karlsruhe, Germany) to allow axial scanning of the sample.

Multiphoton fluorescence was collected and collimated by the same microscope objective, sent back through the beam scanner, passed through the dichroic beamsplitter and a BG39 (Schott AG, Mainz, Germany) filter that further blocked the laser light, and then focused onto a photomultiplier tube (H6780, Hamamatsu Photonics, K. K., Hamamatsu City, Japan). Motion of the beam-scanner and the piezoelectric translation stage was coordinated with data acquisition using a National Instruments NI-DAQ 6259 data acquisition device (National Instruments, Austin, TX, USA) and custom software written in the Labview (National Instruments) environment. The focused beam diameter (at the 1/e intensity) for the 1.2 NA objective is 420 nm. The number of pixels acquired per scan line was chosen to significantly over-sample the data. Image stacks were acquired with an axial separation of 250 nm between subsequent frames. Appropriate cell divisions to scan using multiphoton microscopy were selected using conventional wide-field epi-fluorescence microscopy, available in the same microscope. A pco.1600 CCD camera (PCO AG, Kelheim, Germany) was used to display the fluorescence image; the focused laser spot was also visible in the camera image. BG39 filters were used to ensure that no laser light was visible through the eyepieces of the microscope.

### Analysis of multiphoton cross sectioning data

The software for determining the relative amount of fluorescence in the ana-telophase poles was written in Matlab (The Mathworks, Inc., Natick, Massachusetts, USA). First, the user defined the region surrounding each chromosome pole in the cell division for each of the images in the stack. The program then determined the number of pixels within each region that were above a given threshold and summed this for all images in the stack. For each value of the threshold, the ratio of the amount of fluorescence in each region to that in the first region was calculated. A mean threshold was calculated by averaging the results for all thresholds; the standard deviation of these results was used to calculate the uncertainty in the ratios.

### Real time microscopy

Time-lapse images were obtained on a Zeiss LSM 510 META system with an inverted Zeiss Axiovert 100 M microscope and LSM 510 META software version 3.2 (Carl Zeiss, Jena, Germany). GFP was excited with the 488 nm line of a krypton-argon laser and emission was collected through a Plan-Fluar 100x/1.45 NA objective using a band-pass 505–550 nm filter. Live-cell images were recorded every 60 s through a Plan-Neofluar 40x/0.75 NA objective. The scan speed was 1.5 ms per line pair. The pinhole was adjusted to 1 AU (Airy units) when acquiring confocal images and set to 5 AU or above for live-cell series.

## Supporting Information

Movie S1 Bipolar cell division in HEK293 monitored at 1 frame/min. The time from initiation of metaphase to initiation of anaphase (M-A) time was 35 min and the time from initiation of anaphase to interphase (A-I) was 32 min.
(79.08 MB CDR)

Movie S2 Multipolar cell division in HEK293 shown from metaphase to interphase; M-A was 36 and A-I 21 min. Chromatid segregation occurs in two distinct steps, resulting in four daughter nuclei.
(35.94 MB CDR)

Movie S3 Multipolar cell division in HEK293 shown from metaphase to interphase; M-A was 88 min and A-I 40 min.
(34.35 MB CDR)

Movie S4 The tetrapolar metaphase plate in Movie S3 results in three daughter nuclei as shown by three-dimensional projection of confocal image stacks.
(25.76 MB CDR)

Movie S5 Multipolar cell division in HEK293 showing transition from a complex metaphase configuration to four daughter nuclei; M-A was 40 min, while A-I 1was 10 min; no clear anaphase configuration was observed.
(59.91 MB CDR)
